# Eukaryotic Initiation Factor 2B (eIF2B) GEF Activity as a Diagnostic Tool for EIF2B-Related Disorders

**DOI:** 10.1371/journal.pone.0008318

**Published:** 2009-12-15

**Authors:** Laetitia Horzinski, Aurélia Huyghe, Marie-Céleste Cardoso, Céline Gonthier, Lemlih Ouchchane, Raphael Schiffmann, Pierre Blanc, Odile Boespflug-Tanguy, Anne Fogli

**Affiliations:** 1 INSERM U931-CNRS 6247- Université Clermont, GReD, Clermont-Ferrand, France; 2 Université Clermont 1, UFR Médecine, Clermont-Ferrand, France; 3 CHU de Clermont-Ferrand, Service de Biochimie Médicale et Biologie Moléculaire, Clermont-Ferrand, France; 4 Université de Clermont, UFR Médecine, EA3295, Equipe de Recherche en signal et Imagerie Médicale, Clermont-Ferrand, France; 5 CHU de Clermont-Ferrand, Hôpital Gabriel Montpied, Laboratoire de Biostatistiques, Télématique et Traitement d'Images, Clermont-Ferrand, France; 6 Institute of Metabolic Disease, Baylor Research Institute, Dallas, Texas, United States of America; 7 CHU de Clermont-Ferrand, Service de Génétique Médicale, Centre de Référence Leucodystrophies, Hôpital Hôtel-Dieu, Clermont-Ferrand, France; Hospital Vall d'Hebron, Spain

## Abstract

**Background:**

In recent years, the phenotypes of leukodystrophies linked to mutations in the eukaryotic initiation factor 2B genes have been extended, classically called CACH/VWM (Childhood ataxia with cntral hypomyélination/vanishing white matter disorder). The large clinical spectrum observed from the more severe antenatal forms responsible for fetal death to milder adult forms with an onset after 16 years old and restricted to slow cognitive impairment have lead to the concept of eIF2B-related disorders. The typical MRI pattern with a diffuse CSF-like aspect of the cerebral white matter can lack particularly in the adult forms whereas an increasing number of patients with clinical and MRI criteria for CACH/VWM disease but without eIF2B mutations are found. Then we propose the use of biochemical markers to help in this difficult diagnosis. The biochemical diagnosis of eIF2B-related disorder is difficult as no marker, except the recently described asialotransferrin/transferrin ratio measured in cerebrospinal fluid, has been proposed and validated until now. Decreased eIF2B GEF activity has been previously reported in lymphoblastoid cell lines from 30 eIF2B-mutated patients. Our objective was to evaluate further the utility of this marker and to validate eIF2B GEF activity in a larger cohort as a specific diagnostic test for eIF2B-related disorders.

**Methodology/Principal Findings:**

We performed eIF2B GEF activity assays in cells from 63 patients presenting with different clinical forms and eIF2B mutations in comparison to controls but also to patients with defined leukodystrophies or CACH/VWM-like diseases without eIF2B mutations. We found a significant decrease of GEF activity in cells from eIF2B-mutated patients with 100% specificity and 89% sensitivity when the activity threshold was set at ≤77.5%.

**Conclusion:**

These results validate the measurement of eIF2B GEF activity in patients' transformed-lymphocytes as an important tool for the diagnosis of eIF2B-related disorders.

## Introduction

Mutations in the *EIF2B1*-*5* genes (OMIM 606686, 606454, 606273, 606687, 603945) encoding the subunits of the ubiquitously expressed eukaryotic initiation factor 2B (eIF2B) have been reported in a group of clinically heterogeneous leukodystrophies termed eIF2B-related disorders [1], [2], [3], [4]. Disease severity is correlated with age at disease onset, with stress onset trigger or aggravating factors [5], [6]. A large clinical spectrum is observed and several distinct forms have been proposed: i) the classical childhood ataxia with central hypomyelination/vanishing white matter disease (CACH/VWM, OMIM 603896), with progressive neurological deterioration between age 2-5 years [1], [3], ii) the infantile severe forms with disease onset <2 years and rapid fatal evolution [7], iii) the most severe antenatal forms responsible for fetal death [8], and iv) the milder forms with disease onset >5 years and restricted to slow cognitive impairment [6], [9].

The typical MRI pattern shows a diffuse CSF-like aspect of the cerebral white matter of the cerebral hemispheres and this pattern recognition permits the selection of patients eligible for the *EIF2B1-5* genes sequencing [10]. But MRI can lack particularly in the adult forms whereas an increasing number of patients with clinical and MRI criteria for CACH/VWM disease but without *EIF2B1-5* genes mutations (CACH/VWM-like) are found, underlining the necessity to have biochemical markers to help in the diagnosis process and in the selection of patients eligible for *EIF2B1-5* direct sequencing.

Two biochemical markers have been recently proposed as potential tools for the screening of eIF2B-related disorders: the decrease of asialotransferrin/total transferrin in CSF [11], [12], and the decrease of the eIF2B GEF activity measured in Epstein-Barr Virus (EBV)-transformed lymphocytes or lymphoblasts (LLB) [13]. In fact, eIF2B is a key regulator of the protein synthesis particularly under cellular stresses through its nucleotide guanine exchange (GEF) activity: it converts the initiation factor 2 (eIF2) from an inactive GDP-bound form to an active eIF2-GTP complex [14]. Measurement of eIF2B GEF activity in patients' LLB has been proposed as a potential diagnostic tool relating to a previous work showing decrease of this GEF activity in LLB from 30 affected eIF2B-mutated patients in comparison to controls. In order to further evaluate the specificity and sensitivity of eIF2B GEF activity in patients' LLB regarding the diagnosis of eIF2B-related disorders, we extended this initial cohort of 30 to 63 mutated patients and compared the results not only to healthy non mutated subjects but also to patients with defined leukodystrophies or CACH/VWM-like diseases.

## Methods

### Objectives

Our hypothesis is that eIF2B GEF activity measurement in LLB is a specific and sensitive marker that would be powerful to select patients eligible for the *EIF2B1-5* genes sequencing and then to help in the molecular diagnosis of eIF2B-related disorders. Our objective is then to extend our initial cohort of 30 [13] to 63 eIF2B-mutated patients and to compare the results to healthy subjects but also to CACH/VWM-like patients, in order to evaluate the specificity and sensitivity of eIF2B GEF activity in patients' LLB regarding the diagnosis of eIF2B-related disorders.

### Participants

The GEF activity was measured in LLB from 63 eIF2B-mutated patients (including 30 patients already reported [13] and 13 patients never reported todate, (Table 1), 18 clinically healthy subjects (controls) and 38 patients with leukodystrophies of other causes, termed eIF2B-unrelated leukodystrophic group (Table 2). This last group included:

- 19 leukodystrophic patients with an identified genetic defect and termed OL (other leukodystrophy)-patients: 9 *GFAP-*mutated patients (with Alexander disease, OMIM 203450), 5 *MLC1*-mutated patients (with megalencephalic leukoencephalopathy with cysts, OMIM 604004) and 5 *PLP1*-mutated patients (with Pelizaeus-Merzbacher disease, PMD, OMIM 312080);
- 19 patients with the presence of clinical and/or MRI features observed in eIF2B-related disorders but screened negative for mutations in the coding regions of the five *EIF2B1-5* genes genes, and termed CACH-VWM-like patients.

**Table 1:** Clinical data and eIF2B GEF activity measured in lymphoblasts from the 63 eIF2B-mutated patients.

Patient^a^	Age at disease onset (y)	Disease evolution (y)	Score of disability^b^	Mutated gene	Amino-acid change^c^	eIF2B GEF activity (%)^d^	% AT^e^
1187-1	57	11	3	*EIF2B3*	p.Ser14Phe/p.Ala87Val	111.6±3.6	NA
1074-1	8	NA	NA	*EIF2B5*	p.Arg113His/p.Arg113His	108±11.2	NA
971-1	5	11	1	*EIF2B3*	p.Glu136Pro/?	105.5±6.8	NA
1135-1	5	1.1	2	*EIF2B5*	p.Arg113His/p.Arg315Cys	99±7.4	NA
*432-1	3	8.2	5	*EIF2B5*	p.Arg113His/p.Trp628X	90.4±1.8	2.11
356-1	3	16	5	*EIF2B4*	p.Pro243Leu/p.Pro243Leu	80.4±2.8	NA
736-1	8	4	1	*EIF2B4*	p.Arg374Cys/p.Arg374Cys	80±0	NA
630-1	4.5	10.5	4	*EIF2B5*	p.Arg113His/p.Arg113His	77.5±2.5	5.50
*370-2	4.5	5	3	*EIF2B5*	p.Arg113His/p.Arg195His	77±2.5	NA
1304-1	18	32	2	*EIF2B5*	p.Arg113His/p.Arg113His	76.1±4.3	NA
1008-1	18	7	5	*EIF2B5*	p.Arg113His/p.Arg113His	76.1±2.6	NA
954-1	3.5	4	4	*EIF2B5*	p.Arg113His/p.Arg269Leu	75.9±1.6	7.77
*76-1	17	8	1	*EIF2B2*	p.Glu213Gly/p.Lys273Arg	75.5±10	NA
299-1	11	17	4	*EIF2B5*	p.Arg113His/p.Arg113His	75.2±5.5	NA
338-1	10	16	5	*EIF2B5*	p.Arg113His/p.Arg113His	75.2±1.5	NA
1407-1	24	2	1	*EIF2B5*	p.Arg113His/p.Arg222Trp	74.9±0.8	NA
*370-1	3.5	7	3	*EIF2B5*	p.Arg113His/p.Arg195His	71.5±9	NA
73-1	4	11	3	*EIF2B5*	p.Arg113His/p.Arg113His	70.8±7	NA
904-1	2	1.5	1	*EIF2B5*	p.Arg113His/p.Gly481fs493X	70±1.2	4.52
1014-1	16	0.5	0	*EIF2B5*	p.Arg113His/p.Arg195Cys	68±4	NA
*470-2	2	4	1	*EIF2B5*	p.Glu81Lys/p.Arg113His	67.9±1.8	NA
1240-1	17	4	0	*EIF2B3*	p.Ala202Thr/p.Arg438X	67.7±2	NA
1627-1	7	24	3	*EIF2B5*	p.Pro87Leu/p.Arg113His	67.1±1.8	NA
*76-2	7	11	3	*EIF2B2*	p.Glu213Gly/p.Kys273Arg	67±5.1	NA
807-1	6	4.5	3	*EIF2B5*	p.Arg113His/p.Arg113His	67±4.3	NA
435-1	3	5.5	3	*EIF2B5*	p.Arg113His/p.Arg422X	67±3	NA
1467-1	25	19	1	*EIF2B5*	p.Tyr483Cys/p.Arg195His	66.3±0.6	NA
1232-1	28	3	1	*EIF2B5*	g.IVS8+59A/G/?	64.3±7.8	NA
*648-2	7	3	1	*EIF2B2*	p.Glu213Gly/p.Glu213Gly	64±4	4.11
1108-1	3.5	1.5	4	*EIF2B5*	p.Arg113His/c.[+2081delG]	63.6±2.3	NA
1115-1	2	6	2	*EIF2B5*	p.Pro427Leu/p.Pro427Leu	63.4±8.2	NA
1441-1	2.8	3.2	4	*EIF2B5*	p.Leu106Phe/p.Arg113His	63.2±2	NA
997-1	7	4	1	*EIF2B5*	p.Arg113His/p.Arg113His	61.3±0.3	NA
*823-1	4	2	4	*EIF2B3*	p.His341Gln/p.His341Gln	61±6	NA
*576-2	7	5	0	*EIF2B5*	p.Tyr343Cys/p.Ile385Val	61±0.2	NA
1012-1	14	10	5	*EIF2B2*	p.Glu213Gly/p.Glu213Gly	60±2	NA
663-1	3.5	1.5	1	*EIF2B5*	p.Arg113His/p.Arg113His	59.9±3.8	NA
1004-1	2	1	1	*EIF2B2*	p.Glu213Gly/p.Glu213Gly	59.9±0.8	NA
*375-2	2.5	3	5	*EIF2B5*	p.Arg113His/p.Glu650Leu	59.4±0.7	NA
*648-1	7	14	4	*EIF2B2*	p.Glu213Gly/p.Glu213Gly	59±1	NA
442-1	2.5	NA	NA	*EIF2B5*	p.Glu81Lys/p.Arg113His	58.8±0.3	NA
308-1	5	8	4	*EIF2B5*	p.Arg113His/p.Arg113His	57.1±6.1	NA
*576-1	8	11	1	*EIF2B5*	p.Val73Gly/p.Arg113His	56±4	NA
359-1	6	1.5	5	*EIF2B4*	p.Pro243Leu/p.Pro243Leu	54±6	NA
522-1	3.5	3.5	3	*EIF2B5*	p.Ala16Asp/p.Arg113His	54±6	4.80
*432-2	2	8.2	5	*EIF2B5*	p.Arg113His/W628stop	53.9±0.9	NA
*570-2	1.5	6.5	4	*EIF2B4*	p.Arg209Gln/p.Arg209Gln	52±3	NA
*470-1	3	4	1	*EIF2B5*	p.Glu81Lys/p.Arg113His	51.5±4.5	NA
984-1	5	4	2	*EIF2B2*	p.Glu213Gly/p.Glu213Gly	51.5±0.5	NA
*571-2	1.2	9	5	*EIF2B5*	p.Phe56Val/p.Arg315His	50±5	NA
928-1	3	5	1	EIF2B5	p.Arg113His/p.Arg113His	49±3	NA
995-1	1	2	4	*EIF2B5*	p.Arg113His/p.Leu425Arg	48±2	NA
*590-2	1	4	5	*EIF2B5*	p.Tyr343Cys/p.Ile385Val	45.8±2.2	4.36
949-1	4	9	4	*EIF2B4*	p.Pro243Leu/p.Pro243Leu	45.6±1.8	NA
*590-1	3	4	3	*EIF2B5*	p.Tyr343Cys/p.Ile385Val	44.9±4.3	NA
357-1	2	2	5	*EIF2B5*	p.Arg136Cys/p.Arg339Trp	44.5±4.5	NA
291-1	1.5	6.5	4	*EIF2B5*	p.Arg113His/p.Arg113His	41.5±6	NA
*571-1	0.8	9	5	*EIF2B5*	p.Phe56Val/p.Arg315His	40±3	NA
569-1	3.5	19.5	4	EIF2B5	p.Arg113His/p.256_281del	40±2	NA
1388-1	22	17	1	*EIF2B5*	p.His214Arg/p.Arg269X	39±7	NA
942-2	6	7	1	*EIF2B4*	p.Cys465Arg/p.Tyr489TThr	34.5±1	NA
137-1	5	7.5	1	*EIF2B2*	p.Glu213Gly/p.Glu213Gly	33.9±3.2	NA
1036-1	0.8	0.5	5	*EIF2B5*	p.Pro323Ser/p.Pro427Leu	30±7.5	NA

a *  =  Familial form: two affected children in the same family; underlined: new patients whose genotype has not been reported in our previous studies [5], [13].

b score of disability as previously described [5]: 0: no neurologic signs, 1: stiff gait, 2: walk with help, 3: wheelchair-bound, 4: help for daily living, 5: death.

c Amino-acid numbers refer to the eIF2B peptide corresponding sequence.?  =  mutation not yet identified.

d Expressed as % control value ± standard deviation (assays performed in triplicate); underlined: new eIF2B GEF activity measured and not reported in [13].

e Asialotransferrin ratio (AT) in % already described [11], [12]. NA: Not available.

**Table 2:** eIF2B GEF activity measured in lymphoblasts from the 38 leukodystrophic patients (19 OL and 19 CACH/VWM-like) and the 18 controls.

Patient	Disease^a^	Mutated gene	Mutation^b^	eIF2B GEF activity (%)^c^
81	PMD	*PLP1*	duplication	108.6±10.4
1437	AD	*GFAP*	p.Asp114Glu	103.8±6.6
940	MLC	*MLC1*	p.Leufs	109.6±0.4
002	PMD	*PLP1*	duplication	98.4±1.3
672	AD	*GFAP*	p.Asn77Tyr	133.6±9
958	AD	*GFAP*	p.Arg407Met	113.2±5.1
150	PMD	*PLP1*	duplication	103.4±4.5
42	PMD	*PLP1*	duplication	86.2±4.4
726	AD	*GFAP*	p.Arg239Cys	119.8±1.6
989	AD	*GFAP*	p.Met74Lys	101.5±8.4
256	MLC	*MLC1*	NA	102.6±4.3
737	AD	*GFAP*	p.Arg88Cys	101±14.8
1047	MLC	*MLC1*	p.Ser289Tyr/?	98.7±13
750-1	AD	*GFAP*	p.Arg79His	105.6±1.8
750-2	AD	*GFAP*	p.Arg79His	79.9±3.9
613	MLC	*MLC1*	NA	101.8±2.5
968	PMD	*PLP1*	duplication	105.9±6.3
1207	MLC	*MLC1*	p.Cys46fs	107.7±5.3
758	AD	*GFAP*	p.Arg88Cys	96.4±4.4
1011-1	CACH-L	-	-	104.43±6.5
1242-1	CACH-L	-	-	105.29±8.8
1469-1	CACH-L	-	-	103.30±1.7
1479-1	CACH-L	-	-	99.75±2.2
1082-1	CACH-L	-	-	89.5±8.8
1196-1	CACH-L	-	-	110.4±3.3
1290-2	CACH-L	-	-	105.9±2.7
1211-1	CACH-L	-	-	87.4±5.1
1200-1	CACH-L	-	-	108.5±2
798-1	CACH-L	-	-	119.0±2.3
1253-1	CACH-L	-	-	107.6±6.6
1206-1	CACH-L	-	-	94.67±8.2
K80	CACH-L	-	-	89.27±2.6
K128	CACH-L	-	-	84.76±1.2
K112	CACH-L	-	-	100.41±2.5
1313-1	CACH-L	-	-	82.02±1.2
1283-1	CACH-L	-	-	101.40±6.8
1454-1	CACH-L	-	-	111±16.6
331-1	CACH-L	-	-	88.3±9.5
N1	C	-	-	100
N2	C	-	-	99
N3	C	-	-	101
N4	C	-	-	100
N5	C	-	-	102
N6	C	-	-	100
N7	C	-	-	98
N8	C	-	-	100
N9	C	-	-	94.3±7
N10	C	-	-	100±0.3
N11	C	-	-	95.3±7.5
N12	C	-	-	88.5±1.5
N13	C	-	-	97.5±2.5
N14	C	-	-	99.5±7
N15	C	-	-	99.3±6.1
N16	C	-	-	93.5±0.5
N17	C	-	-	96.5±0.5
N18	C	-	-	102±2

a OL: PMD: Pelizaeus-Merzbacher disease, AD: Alexander disease, MLC: Megalencephalic Leukoencephalopathy with Cysts; and CACH/VWM-like: CACH-L.

b ?: second mutation not yet identified; NA: not available.

c Expressed as % control value ± standard deviation (assays performed in triplicate); underlined: new eIF2B GEF activity measured and not reported in [13].

### Measurement of eIF2B GEF Activity in Transformed Lymphocytes

The direct GEF activity of the eIF2B complex was measured in triplicate in protein extracts from patients'EBV-transformed lymphocytes (LLB) as already described [13], [15].

### Ethics

An Institutional Review Board of the participating centers (Comité de Protection des Personnes Sud-Est VI, 2009-A00188-49) approved the use of human subjects for this study. A written informed consent was obtained from all patients.

### Statistical Methods

The eIF2B GEF activity was considered as a continuous variable and results are displayed as mean ± SEM (standard error of the mean). Since the normality of eIF2B GEF (assessed by a Kolmogorov-Smirnov test) was not rejected, a one-way analysis of variance (ANOVA 1) was performed to assess the links between GEF activity and the patients'groups. On condition of a significant F-test for the ANOVA 1, a post-hoc multiple comparisons procedure was performed controlling for a 5% family-wise type I error using the Tukey honestly significant difference (THSD) test. A Spearman correlation coefficient (r) was calculated between GEF activity and age at disease onset (correleted to disease severity [5]). The Receiver Operating Characteristic, or ROC curve analysis (graphical plot of the sensitivity *versus* (1-specificity) for a binary classifier system as its discrimination threshold is varied), was performed on MedCalc® (v10.4, Mariakerke, Belgium) to determine the optimal threshold of GEF activity which best discriminates between eIF2B-mutated (n = 63) and eIF2B-unrelated leukodystrophic patients (n = 38), aiming a 100% specificity and the best associated sensitivity. The area under the ROC curve (AUC) was also estimated (with its 95% confidence limits) and tested towards 0.5. All remaining statistical analyses were performed on SAS® (v9.1, Cary, USA) with a type I error set at 5%.

## Results

### eIF2B GEF Activity in 63 eIF2B-Mutated Patients in Comparison to the Other Groups

**Figure 1:**
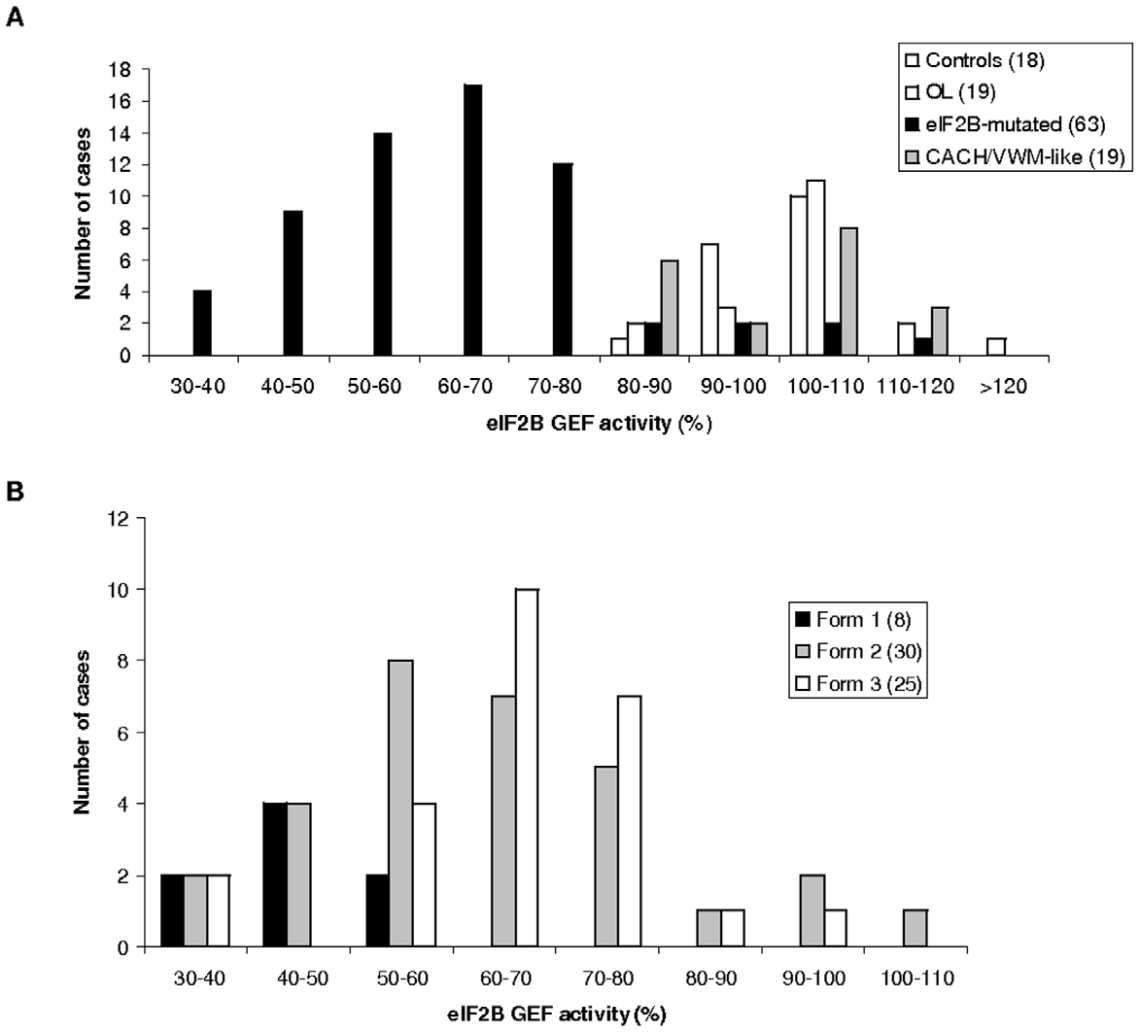
Distribution of patients per classes of eIF2B GEF activity. **A**. Distribution of the patients'groups per classes of eIF2B GEF activity in %. The patients' groups are healthy controls, eIF2B-mutated, others leukodystrophies (OL) and CACH-VMW-like. **B**. Distribution of the 63 eIF2B-mutated patients per classes of eIF2B GEF activity. The mutated patients have been classified into three clinical groups depending of their clinical severity, according to previous studies [5]. Form 1: disease onse before 2 years, form 2: disease onset berween 2 and 5 years, form 3: disease onset >5 years.

**Figure 2:**
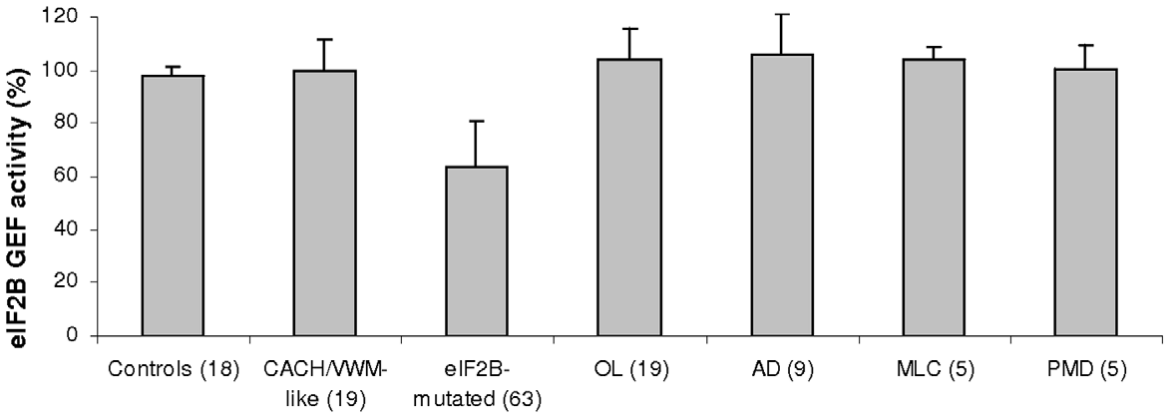
Decreased eIF2B GEF activity in eIF2B-mutated patients' lymphoblasts (LLB). The eIF2B GEF activity was measured in LLB from 63 eIF2B-mutated patients in comparison to 8 healthy controls, 19 patients carrying other leukodystrophies (OL: 9 patients with Alexander disease, AD, 5 patients with Pelizaeus-Merzbacher disease, PMD, and 5 patients with Megalencephalic leukoencephalopathy with cysts, MLC), and 19 CACH/VWM-like patients without eIF2B mutations. Experiments were carried out in triplicate. Data are presented as percentage of exchange activity of control LLB. The statistical multiple comparisons analysis between all the seven groups showed significant differences (*) only for the eIF2B-mutated group (p<0.001).

The eIF2B GEF activity was measured in LLB of 63 affected patients exhibiting various eIF2B mutations, age of onset and disease severity (Table 1). Despite the wide range of GEF activity in eIF2B-mutated cells (63.2±17.1%, range: 30–111.6%) (Figure 1A), multiple comparisons demonstrated a significant difference between the group of eIF2B-mutated patients (n = 63) and the three other groups of patients [OL-patients (104.1±11.4%, range: 80–133.6%, n = 19), CACH/VWM-like (99.6±10.3%, range: 82–119%, n = 19) and controls (98±3.3%, range: 88.5–102%, n = 18)] (F(3,115)  = 71.1, p<0.001) (Tables 1 and 2, Figures 1A and 2). The OL, CACH/VWM-like and controls groups had similar GEF activity (THSD test). The THSD test further detailed that only the eIF2B-mutated group had significantly lower GEF activity compared with each of the three other groups (OL, CACH/VWM-like and controls).

Pooling CACH/VWM-like patients and OL-patients (group of eIF2B-unrelated leukodystrophic patients, n = 38), the aforementioned comparison of GEF activity led still to a significant result (F(2,116)  = 106.1, p<0.0001) and the THSD test showed again the same significantly lower GEF activity in the eIF2B-mutated group as compared with each of the two other groups (eIF2B-unrelated leukodystrophic and controls). Then difference in GEF activity remained statistically significant between eIF2B-mutated patients and the other groups tested.

### Correlation between Age at Disease Onset and GEF Activity

A weak correlation was found between GEF activity measured in the eIF2B-mutated LLB and age at disease onset (r = 0.4309, p = 0.0004). Two patients 356–1 and 432–1 (Table 1) exhibited high GEF activities (respectively 80.4±2.8% and 90.4±1.8%) despite a classical clinical form (disease onset at 3 years followed by severe disability within 3 years and death occurring after 16 years in one patient). On the other hand, patient 1388–1 affected with an adult onset, slowly progressive form (only stiff gait after 17 years of disease progression), exhibited a low GEF activity (39±7%) (Table 1). However, the eight patients with onset ≤2 years (disease severity form 1) always had eIF2B GEF activity <55% (45.1±7.8%, mean age at disease onset: 1.2 year) (Table 1B). The correlation coefficient is higher in this group of eight patients (r = 0.68, p = 0.06) compared to the 30 patients carrying the clinical severity form 2 with onset between 2 and 5 years (r = 0.11, p = 0.52), and to the 25 patients carrying the severity form 3 with onset >5 years (r = 0.45, p = 0.0218) (Table 1B).

### ROC Curve Analysis of eIF2B GEF Activity in the Leukodystrophic Groups

**Figure 3:**
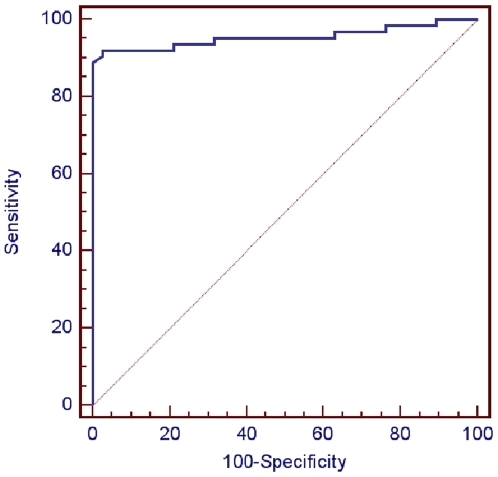
ROC (Receiver Operating Characteristic) curve of eIF2B GEF activity regarding the diagnosis of eIF2B-related disorders. The ROC curve analysis was performed to determine the optimal threshold of GEF activity which best discriminates between eIF2B-mutated (n = 63) and eIF2B-unrelated leukodystrophic patients (n = 38). The ≤77.5% threshold achieves 100% specificity (95% CL = 90.7–100%) and 88.9% sensitivity (95% CL = 78.4–95.4%). The area under the ROC curve (AUC)  = 0.955; standard error  = 0.0244; 95% confidence interval: 0.894 to 0.986; test for the: AUC  = 0.5, p = 0.0001.

Three out of the 63 eIF2B-mutated patients (4.8%) had GEF activity >100%. They expressed a mild juvenile/adult form of the disease with onset ≥5 years of age (Table 1). The ROC curve analysis of eIF2B GEF activity performed towards differential diagnosis between eIF2B-related (n = 63) and eIF2B-unrelated (n = 38) leukodystrophy patients lead to a pathognomonic threshold “≤77.5% of GEF activity”, achieving 100% specificity (95% CL = 90.7–100%) and 88.9% sensitivity (95% CL = 78.4–95.4%), with an almost perfect discrimination (AUC = 0.96±0.024, p<0.0001) (Figure 3). Only 7/63 (11.1%) eIF2B-mutated patients had GEF activities >77.5%. These patients presented with classic to milder clinical forms with onset ranging from 3 to 57 years, slow disease progression, and carrying mutations in different *EIF2B1-5* genes (Table 1). This group illustrates that the high level of GEF activity found is not linked to the type of mutated gene nor to a specific degree of clinical severity. However, none of these patients had a disease onset <3 years.

## Discussion

Analysis of this extended cohort showed that eIF2B GEF activity measured in patients' LLB distinguishes eIF2B-mutated patients from those with eIF2B-unrelated leukodystrophies with 100% positive predictive value (PPV) and 89% negative predictive value at ≤77.5% threshold. At this threshold, the assay systematically excludes patients without eIF2B mutations. Therefore, it represents an interesting screening tool to select patients for a direct sequencing of the *EIF2B1-5* genes. For leukodystrophic patients with >77.5% GEF activity, the probability to find eIF2B mutations is 15% (7/45) and increases to 26.9% (7/26) if patients have clinical and/or MRI features typical to eIF2B-related disorder. Therefore, *EIF2B1-5* sequencing is still indicated for patients who are clinically suspected of eIF2B-related disorder with GEF activity >77.5%, particularly in milder forms.

The wider range of disease onset reported in this cohort in comparison to previous work [13] may explain the weaker correlation found between age at disease onset and GEF activity. Such correlation is better in severe forms with GEF activity <55% for patients with onset <2 years (Figure 1B). Discrepancies in the GEF activity values were found among patients of the same group of disease onset or with the same mutations such as siblings 432–1 and 432–2 (GEF activities at respectively 90% and 53.9%), confirming that GEF activity in LLB is modulated by factors other than eIF2B mutations.

Determination of the asialotransferrin/total transferrin ratio in CSF is also a reliable marker to distinct eIF2B-mutated from non-mutated patients with 100% sensitivity and 94% specificity associated to 8%-ratio threshold [12]. This marker has been determined in parallel for five of our 63 eIF2B-mutated patients and a decreased ratio has been found in CSF (Table 1) in all five, including patient 432–1 with a surprisingly normal eIF2B GEF activity (90.4±1.8%) compared to his sibling. This suggests that these two biomarkers, if available for the same patient, may be complementary in order to assess with 100% sensitivity, specificity and PPV.

### Limitations

Limitations of this study are:

- the relatively small number of patients overall, as eIF2B-related disorders are rare disorders;
- the age of disease onset, that may be imprecise for some patients, since it is determined retrospectively.
